# Reliability and validity of the RS14 in orphaned and separated adolescents and youths in western Kenya

**DOI:** 10.1371/journal.pone.0241699

**Published:** 2020-11-24

**Authors:** Sarah C. Sutherland, Harry S. Shannon, David Ayuku, David L. Streiner, Olli Saarela, Lukoye Atwoli, Paula Braitstein

**Affiliations:** 1 Division of Epidemiology, Dalla Lana School of Public Health, University of Toronto, Toronto, Canada; 2 Department of Health Research Methods, Evidence, and Impact, Faculty of Health Science, McMaster University, Hamilton, Canada; 3 Institute for Work & Health, Toronto, Canada; 4 Department of Behavioral Sciences, School of Medicine, College of Health Sciences, Moi University, Eldoret, Kenya; 5 Department of Psychiatry and Behavioural Neurosciences, McMaster University, Hamilton, Canada; 6 Department of Psychiatry, University of Toronto, Toronto, Canada; 7 Department of Mental Health, School of Medicine, College of Health Sciences, Moi University, Eldoret, Kenya; 8 Department of Epidemiology and Medical Statistics, School of Public Health, College of Health Sciences, Moi University, Eldoret, Kenya; 9 Academic Model Providing Access to Healthcare (AMPATH), Eldoret, Kenya; 10 Department of Medicine, School of Medicine, College of Health Sciences, Moi University, Eldoret, Kenya; 11 Regenstrief Institute, Inc, Indianapolis, Indiana, United States of America; Vilnius University, LITHUANIA

## Abstract

**Purpose:**

The 14-item Resilience Scale (RS14) is a tool designed to measure psychological resilience. It has been used effectively in diverse populations. However, its applicability is largely unknown for Sub-Saharan adolescent populations and completely unknown for orphaned and separated adolescents and youths (OSAY), a highly vulnerable population for whom resilience may be critical. This study assesses the RS14’s psychometric properties for OSAY in Uasin Gishu County, Kenya.

**Methods:**

Survey responses from a representative sample of 1016 OSAY (51.3% female) aged 10–25 (mean = 16; SD = 3.5) living in institutional and home-based environments in Uasin Gishu County were analyzed. The RS14’s psychometric properties were assessed by examining internal consistency reliability, confirmatory factor analyses, and convergent validity using correlations between resilience and each of social support and depression. Sub-analyses were conducted by age and sex.

**Results:**

Resilience scores ranged from 14–98 (mean = 66; SD = 19) with no sex-based significant difference. Resilience was higher for those aged ≥18 (mean = 69; range = 14–98) versus age <18 (mean = 65; range = 14–98). Internal consistency was good (Cronbach’s α = .90). Confirmatory factor analysis indicated a 1-factor solution, though the model fit was only moderate. Resilience was positively correlated with social support in all ages (.22; *p* < .001) and negatively correlated with depression in individuals age <18 (-.22; *p* < .001). The relationship between resilience and depression in individuals age ≥18 was statistically significant only in females (-.17; *p* = .026).

**Conclusion:**

This study demonstrates reasonable evidence that the RS14 is both valid and reliable for measuring psychological resilience in the population of OSAY in western Kenya.

## Introduction

Resilience refers to an individual’s ability to recover from and adapt in the face of social disadvantage and highly adverse conditions [1]. It is also a psychological trait which has gained clinical relevance and prominence in health research. It has been described as the capacity of an individual to regain balance and persevere despite challenges, and is closely linked to confidence in one’s own abilities. Resilience is considered protective against various mental health disorders including depression [2].

While early research considered resilience to be a relatively stable personality trait, newer studies have demonstrated that it can be developed over time via coping techniques that allow an individual to navigate crises effectively, maintaining optimism and balancing negative emotions with positive ones [1, 3]. Positive emotions are strongly linked to resilience through their role in promoting problem solving and flexibility in thinking, as well as counteracting the physiological effects of negative emotions and helping individuals to recover from stressful encounters. Positive emotions are also linked to adaptive coping, creating enduring social resources, and enhancing personal well-being [4, 5]. Age and gender, along with modifiable factors including participation in activities and the presence of supportive relationships, have likewise been associated with resilience and mitigating the negative effects of adverse life situations [6–8]. Social support has demonstrated positive associations with psychological resilience across a variety of populations including in children and adolescents [9]. Developmentally, adolescence is a strategic time for optimizing future mental and physical health as negative behaviour patterns are not yet set. It is a critical stage to develop resilience and positive coping mechanisms that optimize an individual’s ability to achieve success in life.

Many tools measure psychological resilience. These include the Baruth Protective Factors Inventory, Brief Resilience Coping Scale, Brief Resilience Scale, Connor-Davidson Resilience Scale, Resilience Scale for Adults, and the Resilience Scale developed by Wagnild and Young [1]. Several reviews comparing commonly used instruments have found the Wagnild and Young Resilience Scale and its 14-item short form, the RS14, to be the best instruments to use in adolescent populations [1]. While other tools demonstrate acceptable psychometric properties in certain populations, the literature found a general lack of evidence of appropriateness for use with adolescents, or in diverse populations, and a limited number of high-quality studies using these tools [1]. In contrast, the RS and RS14 demonstrated good psychometric properties, ease of use, and an extensive history of validation and use in diverse, international populations including with adolescents and in several languages [1, 10]. The RS14 has undergone successful validation studies including young adults in Japan, Finland, and Lithuania, and specifically in youth and young adult populations or subgroups in Nigeria, Italy, Brazil, Poland, and Portugal, among others [2, 11–17]. An additional benefit of the RS14 is brevity, reducing participant burden by requiring only 3–4 versus 5–7 minutes to complete while still capturing the five essential resilience characteristics and maintaining a strong correlation with the 25-item Resilience Scale [2, 18].

Understanding psychological resilience and its impact on health related outcomes may offer important insight into how to mitigate the effects of adverse life events on vulnerable populations. While resilience has been studied extensively in developed countries, relatively little research has focused on developing countries including Kenya. Resilience studies in Sub-Saharan African (SSA) children and youths are primarily qualitative with modest sample sizes and little between-study consistency in resilience tools [19]. In SSA the RS14 has only been validated in Nigerian adults; never in orphaned and separated adolescents and youths (OSAY) [13].

SSA is home to approximately 50 million OSAY and this number is growing, due in large part to the sub-continent accounting for nearly 68% of worldwide individuals currently living with HIV [20, 21]. As of 2018, approximately 11 million OSAY in SSA, including 2.3 million in Kenya, are thought to have lost one or both parents to AIDS [21]. Compared to non-orphans, OSAY are at higher risk of many negative health behaviours and outcomes including participation in exchange or survival sex, drug and alcohol use, and significant intra-household discrimination, and the experience of physical and sexual violence [22–24]. Psychological, environmental, and social factors, rather than orphan status alone, may influence an individual’s likelihood of engaging in high risk behaviours which put them at risk for HIV infection and other adverse outcomes [22]. Of these factors, psychological resilience is theorized to provide a strong protective effect and there is an urgent need for research into the determinants of resilience among OSAY [25, 26]. The aim of this study was to validate the RS14 instrument for measuring psychological resilience in the Kenyan OSAY population through exploring the instrument’s internal consistency, convergent validity, and checking the unifactoral structure found in previous studies.

## Methods

### Study setting

#### Procedure and participants

The Orphaned and Separated Children’s Assessments Related to Their Health and Well-Being (OSCAR) (R01HD060478) longitudinal cohort study began in 2010. It is designed to investigate the effects of care environment on the physical and mental health of OSAY ≤18 years old at baseline in UG, Kenya, through annual surveys. These include a clinical encounter (including HIV testing) for all participants and a psychosocial encounter for OSAY aged ≥10. The psychosocial encounter is self-administered with assistance from an on-site psychosocial counselor available on request.

Data were obtained from 1016 of 1231 unique participants of Phase II. Exclusions were based on age, as the RS14 is not administered to OSAY aged <10 (n = 27), incomplete RS14 (n = 187), and residing in an ineligible living environment of self-care at the time of data collection (n = 1).

We used the baseline data of Phase II, which began in 2015. Data cleaning occurred first in the field and discrepancies or missing information were verified with the participant on site. This cohort has been described in detail previously [27].

#### Human subjects protection

This study protocol and the parent OSCAR study were approved by the Research Ethics Board at University of Toronto, the Institutional Research Ethics Committees of Moi University College of Health Sciences and Moi Teaching and Referral Hospital, and Indiana University’s Institutional Review Board. Informed consent was provided by the head of each household for family-based care settings (FBS) or Director of the Charitable Children’s Institution (CCIs, i.e. orphanage) for government run institutions. All individuals ≥10 years old provided written assent. When individuals were unable to sign their names, fingerprints were used. All psychosocial assessments were reviewed by a project psychologist for red flags on suicidality, markers of ongoing, active abuse, or if the child was likely to pose a threat to themselves or others. OSCAR study staff followed up on these cases.

### Instruments

#### RS14

The RS14 was included in OSCAR Phase II. It was licensed effective date June 30, 2017 and administered in the original English. The RS14 is strongly correlated with the RS-25 (r = .97). Both instruments center on the ‘Resilience Core’, a set of 5 essential characteristics that constitute resilience: equanimity, authenticity, perseverance, purpose, and self-reliance. Equanimity is defined as having a balanced perspective and the ability to adapt to life changes. Authenticity is the realization that every individual has a unique perspective and must face certain experiences alone. Perseverance is the ability to continue forward despite setbacks. Purpose is the belief that one’s life has innate meaning. Self-reliance is the ability to understand and accept one’s own capabilities and limitations leading to self-efficacy and problem solving.

Each item uses a Likert scale ranging from 1 (strongly disagree) to 7 (strongly agree). Total scores range from 14–98, with ≤64 considered low, 65–81 moderately low to moderate, and ≥82 high levels of resilience. In previous studies, resilience scores generally demonstrate a negative skew with means ranging from 63–87. In the OSCAR study, the response format was simplified from a single-line to individually labeled checkboxes in two columns with responses including ‘Strongly Disagree’, ‘Disagree’, ‘Somewhat Disagree’, ‘Don’t Know’, ‘Somewhat Agree’, ‘Agree’, ‘Strongly Agree’, and ‘Refuse to Answer’. ‘Don’t Know’ was interpreted as a neutral response with a value of 4.

The RS14 is considered internally consistent in many populations with a Cronbach’s α of .76-.96. In a wide range of studies, the RS14 has demonstrated construct validity through content analysis, known groups, correlation studies, factor analysis, convergent/discriminant studies, and pre/post-test intervention studies. While the original RS-25 has been found to have a two-factor structure with subscales including personal competence (17 items) and acceptance of self and life (8 items), most studies of the RS14 have found a single-factor solution to provide the best fit [28]. Despite this, questions in the RS14 draw directly from the five subtypes of resilience and several studies have demonstrated multi-factor solutions to be most appropriate [28]. For this reason both 1-factor and 5-factor solutions were examined.

### 12-item Multidimensional Scale of Perceived Social Support (MSPSS) (social support)

Social support was measured using the MSPSS which aims to measure an individual’s perceived adequacy of social support arising from family, friends, and significant others. These form the instrument’s three subscales. The MSPSS has been found to be psychometrically sound, with strong internal reliability (Cronbach’s α ranging from .86-.90) and factorial validity in a number of diverse populations including South African children and adolescents [29–31].

The MSPSS consists of 12-items that use a 7-point Likert scale ranging from 1 (very strongly disagree) to 7 (very strongly agree) [32]. Scores are added for each subscale, or the entire instrument, and divided by the number of items. Mean scores or categories of level of social support may be used [33]. In the OSCAR survey, original format was replaced by individual checkboxes with responses, values ranging from 1–5, including ‘Strongly Disagree’, ‘Somewhat Disagree’, ‘Don’t Know’, ‘Somewhat Agree’, ‘Strongly Agree’, and ‘Refuse to Answer’. For the purposes of this study, ‘Don’t Know’ was interpreted as a neutral response with a value of 3. Sensitivity analyses were run removing the ‘Don’t Know’ response option, creating a value range of 1–4 for each question. Social support was considered as a continuous measure.

#### Child depression inventory short-form

Depression was measured using the Child Depression Inventory short form (CDI-S) in participants <18 years of age. Due to its easy readability, brevity, and strong psychometric properties, the long form Child Depression Inventory is the most frequently used tool for measuring symptoms of depression in children [34]. The CDI-S distills the original 27-item instrument to 10-items, reducing assessment time and burden. The CDI-S has no subscales. It has sound psychometric properties and is considered comparable with the long form, with strong internal reliability and convergent validity [34]. In 2015, a meta-analysis of 22 studies found the mean Cronbach’s α of the CDI-S was .77 (95% CI = [.74,.80]) [35]. The CDI-S has been used successfully in several populations including children and adolescents orphaned by AIDS in South Africa [36].

For each item, respondents are asked to choose one of three statements that best represents their feelings over the last two weeks. Items are scored on a 3-point scale based on severity of symptoms.

#### PHQ-9

Depression was measured using the 9-item Patient Health Questionnaire (PHQ-9) in participants age 18 years and older. The PHQ-9 is the depression specific module from the full form Patient Health Questionnaire which assesses 8 potential diagnoses. The PHQ-9 aims to aid in criteria based diagnosis of depressive disorders and measure depression severity using questions about the respondent’s feelings over the last two weeks [37]. The PHQ-9 has been found to be psychometrically sound, with Cronbach’s α ranging from .80-.89 and strong convergent validity [38, 39]. The PHQ-9 has been validated in many populations including Kenyan cancer patients (Swahili translation) [38].

Each PHQ-9 item uses a 4-point Likert scale from 0 (not at all) to 3 (nearly every day). Depression severity is interpreted using summary scores, none-minimal = 0–4, mild = 5–9, moderate = 10–14, moderately severe = 15–19, and severe = 20–27 or as a continuous variable for assessing the severity of depressive symptoms [39]. For this study, the PHQ-9 was used as a continuous variable.

### Statistical analysis

Psychometric properties of the RS14 were assessed by examining internal consistency, confirmatory factor analyses, and convergent validity. Internal consistency was evaluated using Cronbach’s α with α ≥.80 considered acceptable [15]. Construct validity was assessed through confirmatory factor analysis using a maximum likelihood fitting procedure. Both a 5-factor and 1-factor solution were evaluated. Measures of model fit included goodness of fit (GFI), adjusted goodness of fit (AGFI), and comparative fit index (CFI) (acceptable fit ≥.90), the root mean square error of approximation (RMSEA) (good fit < .05, acceptable fit .05-.08), standardized root mean square residual (SRMSR) (good fit < .05, acceptable fit .05–.10), and a *χ*^2^ test [40]. Convergent validity was assessed through correlation with depression and social support. For all analyses, *p*-values of < .05 were considered statistically significant. Sub-analyses, stratified individually by gender and age (<18 vs. ≥18 years of age), tested whether the structure and psychometric properties of the RS14 differed between these populations. Sensitivity analyses were conducted to evaluate changes in the psychometric properties of the RS14 and MSPSS based upon the removal of the ‘Don’t Know’ response option. All analyses used two-tailed tests and were conducted using SAS 9.1 [41].

## Results

The sample consisted of 1016 OSAY living in UG, Kenya, aged 10–25 years (mean = 16; SD = 3.5), with 65% of individuals <18 years of age and 35% age ≥18 years [Table 1]. The sample included 51% females (n = 521) and 49% males (n = 495). Sex distribution did not vary significantly by age. A third of participants lived in CCIs while 67% lived in FBS.

**Table 1:** Sample description.

Variable	Age ≤17	Age ≥18	Total
	# (%); Mean (SD; Range)	# (%); Mean (SD; Range)	# (%); Mean (SD; Range)
Total	656	360	1016
Sex			
Female	327 (50)	194 (54)	521 (51)
Male	329 (50)	166 (46)	495 (49)
RS14^a^	65 (19; 14–98)	69 (19; 14–98)	66 (19; 14–98)
CDI-S^b^	2.8 (3.0; 0–14)	-----	-----
PHQ-9^c^	-----	4.3 (4.7; 0–24)	-----
12-item MSPSS^d^	4.2 (0.8; 1–5)	4.4 (0.8; 1–5)	4.3 (0.8; 1–5)

a *14-item Resilience Scale*.

b *Child Depression Inventory short form*.

c *9-item Patient Health Questionnaire*.

d *12-item Multidimensional Scale of Perceived Social Support*.

* p < .05; ** p < .01; *** p < .001.

Resilience scores ranged from 14–98 with a mean of 66 (SD = 19), considered moderately resilient. Depressive symptom scores in individuals <18 years of age (CDI-S scores) ranged from 0–14 (mean = 2.8; SD = 3.0). In individuals ≥18 years of age, PHQ-9 scores ranged from 0–24 (mean = 4.3; SD = 4.7), considered minimal to mild. Social support scores ranged from 1–5 (mean = 4.3; SD = 0.8). Resilience score, social support, and CDI-S score in individuals age <18 did not vary by sex; however, in individuals ≥18 years of age females demonstrated higher PHQ-9 scores than males (mean = 4.8 vs. 3.7). Individuals ≥18 years of age demonstrated higher levels of both resilience (mean = 69 vs. 65) and social support (mean = 4.4 vs. 4.2) as compared to younger individuals [Table 1].

The RS14’s Cronbach’s α was .90. When restricted by subgroup, Cronbach’s α was .90 for males and individuals age <18, and .91 for females and individuals ≥18 years of age, thus showing high internal consistency overall and by age and sex.

Neither the 1-factor nor 5-factor model in the confirmatory factor analysis met all conventional criteria for a good fit (1-factor: GFI = .87; AGFI = .83; CFI = .88; RMSEA = .10; SRMSR = .06; 5-factor: GFI = .89; AGFI = .83; CFI = .90; RMSEA = .10; SRMSR = .06). While each measure approaches the threshold values for a good model fit, only the SRMSR values and the 5-factor model CFI are considered in an acceptable range for good model fit. A *χ*^2^ test demonstrated a statistically significant difference between the observed and expected matrices [Table 2]. The 1-factor model explained 45% of total variance and all factor loadings were ≥.44. The 5-factor model explained 70% of total variance, all factor loadings were ≥.47, and each factor loaded between 2 and 5 items, with 3 factors loading ≤2 items each.

**Table 2:** Confirmatory factor analysis of the RS14 and goodness-of-fit indexes.

Model	Χ^2^ / *df*	CFI ^a^	GFI ^b^	AGFI ^c^	RMSEA ^d^	SRMSR ^e^
Acceptable values	p < .05	≥.90	≥.90	≥.90	< .05 (good)	< .05 (good)
					.05 - .08 (acceptable)	.05 - .08 (acceptable)
1-factor	783.52 / 77 ***	.88	.87	.83	.10	.06
5-factor	690.63 / 67 ***	.90	.89	.83	.10	.06

a *Comparative Fit Index*.

b *Goodness-of-Fit Index*.

c *Adjusted Goodness-of-Fit Index*.

d *Root Mean Square of Approximation*.

e *Standardized Root Mean Square Residual*.

* p < .05; ** p < .01

*** p < .001.

Resilience was shown to have a negative correlation with depression in individuals age <18 (-.22; *p* < .001) and a positive correlation with social support (.22; *p* < .001) in all age groups. These relationships remained statistically significant across age and sex. The relationship between resilience and depression in individuals age ≥18 was not statistically significant (Total:-.05; *p* = .328; Men: .08; *p* = .297), except in women where a negative correlation was found (-.17; *p* = .026) [Table 3].

**Table 3:** Evaluation of convergent validity: Pearson correlations between the RS14^a^ total score and CDI-S, PHQ-9, and 12-item MSPSS.

Variable	Age ≤17	Age ≥18	Female	Male	Total
	PCC^e^ (*p*-value)	PCC (*p*-value)	PCC (*p*-value)	PCC (*p*-value)	PCC (*p*-value)
Depressive symptoms (CDI-S)^b^	-.22 (< .001***)	-----	-.21 (< .001***)	-.23 (< .001***)	-----
Depressive symptoms (PHQ-9)^c^	-----	-.05 (.328)	-.17 (.026*)	-.08 (.297)	-----
Social support (12-item MSPSS)^d^	.22 (< .001***)	.20 (< .001***)	.24 (< .001***)	.20 (< .001***)	.22 (< .001***)

a *14-item Resilience Scale*.

b *Child Depression Inventory short form*.

c *9-item Patient Health Questionnaire*.

d *12-item Multidimensional Scale of Perceived Social Support*.

e *Pearson Correlation Coefficient*.

* p < .05; ** p < .01

*** p < .001.

All sensitivity analyses demonstrated no significant change in results when neutral categories were removed from the resilience and social support variables.

## Discussion

This study confirmed that with strong to moderate psychometric properties the RS14 is both reliable and valid for use in the Kenyan OSAY population.

There was a moderately-low to moderate resilience level (mean = 66). This is higher than the Japanese mean score (64) but lower than those in Poland (73), Nigeria (74), Finland (76), Italy (76), and England (76) [2, 11, 13, 14, 16, 18]. This may be due in part to the relatively young age of study participants as compared to other populations in which the RS14 has been used. The mean level of resilience was higher in older OSAY, comparable to findings in Brazil, Finland, and the Netherlands, though this difference was not statistically significant in the Polish or Italian populations [11, 14–16, 42]. An increase with age is consistent with the theory that resilience develops over time, with older individuals having had more experience recovering from challenges and developing adaptive coping strategies [42]. Consistent with previous studies on the RS14, resilience did not vary by sex [11, 14–16]. While other studies have found associations between resilience and sex, alternate definitions, such as in Bonanno et al. which defined resilience as having 1 or 0 posttraumatic stress disorder symptoms, or tools, such as the 25-item Resilience Scale, were used [6, 12, 13, 42–44].

With a Cronbach’s α of .90, the RS14 demonstrated good internal consistency, similar to that found in other populations [2, 11, 13, 14, 16, 18]. The internal consistency did not vary significantly with age or sex.

While both solutions approached but did not meet the criteria for a good fit, the confirmatory factor analysis demonstrated that a 1-factor solution with all items explained by a common latent factor, resilience, was more appropriate than a 5-factor solution for the RS14 in the primary population and all sub-populations. In this model, all item loadings were ≥.44. A goodness-of-fit assessment demonstrated similar values when comparing 1-factor and 5-factor solutions; however, while in the 5-factor model CFI and GFI were slightly higher (.90 vs. .88; .89 vs. .87), several factors failed to include more than 1–2 items. A unifactoral solution with a moderate fit is more appropriate and parsimonious, with little improvement gained through including additional factors. The unifactoral solution was most appropriate across all age and sex subgroups. Future research including exploratory factor analysis is required to determine the reason for a fair / fair-to-good model fit in this population; however, both internal consistency and convergent validity testing still support the use of the RS14 in Kenyan OSAY.

Resilience demonstrated a positive correlation of .22 with social support, the direction and magnitude of which did not vary significantly by age or sex. This is consistent with previous studies including Nishi et al. which found a correlation of .38 between resilience and perceived number of social supports and a correlation of .12 between resilience and satisfaction with social supports [2]. It is theorized both that social support may improve individual resilience by providing resources to buffer stressful life events and that resilient individuals may be better equipped to gather social resources and enhance their own social support systems [2, 9].

In individuals <18 years of age, the correlation of -.22 (*p* < .001) between resilience and CDI-S score is consistent with both the theory that resilience mitigates an individual’s susceptibility to depression and the inverse relationships between resilience and depression in previous studies [2, 13, 15, 28]. In young adults, ≥18 years old, the overall relationship between resilience and PHQ-9 score was not statistically significant at α = .05, except in females where a correlation of -.17 (*p* = .026) was observed. In this population, females showed significantly higher, and more varied, levels of depressive symptoms as compared to males, with mean scores of 4.8 vs. 3.7 (*p* = .018). Relative homogeneity in depressive symptom scores in men may have limited the ability to detect a significant difference in this population; however, this does not account for the slight positive, if statistically non-significant, relationship with resilience. This may be due in part to a combination of cultural stigma surrounding mental health in Kenya, traditional masculine social roles which limit the socially acceptable expression of illness behaviour, and the gendered nature of help seeking behaviour [45]. Previous research has demonstrated an increase with age in stigmatizing attitudes toward individuals with depressive symptoms, with adolescent males indicating higher levels of stigmatizing attitudes than younger males or adolescent females [46]. Additionally, in a study of peer acceptance of individuals with mental health problems, as age increased males demonstrated decreasing acceptance of other males with depressive symptoms [47]. As such, internalized negative attitudes toward mental health problems may be related to lower and more homogeneous depressive symptom scores in older males. Age based differences may also be due in part to the use of different tools to measure depressive symptoms in OSAY ≤17 and ≥18 years of age.

This study has several important limitations. The MSPSS was adapted with the removal of two of seven response categories: ‘Very Strongly Agree’ and ‘Very Strongly Disagree’. While this improved ease of administration it also limited the use of categorical scoring options and direct comparison of results with previous studies. The use of social support as a continuous measure was not impaired. This compression of response range may also account for the highly significant but lower in magnitude than expected correlations observed between resilience and social support.

The use of different, age-specific, tools to assess depression symptoms in individuals ≤17 and ≥18 years of age limits the interpretation and generalizability of results.

Convergent validity was assessed through the individual correlations of resilience with depression (negative) and social support (positive). While these associations are well documented, the inclusion of additional constructs, such as optimism or post-traumatic symptoms, would have strengthened the generalizability of the conclusions.

Missing data may be non-random and could affect internal and external validity. To minimize bias due to missing data and improve sensitive information accuracy, psychosocial assessments were self-administered, unless assistance was requested, and confidentiality was assured. Surveys were checked for completeness on site, with immediate follow-up to fill incomplete questions. Thus, OSCAR study’s data may not suffer as much underreporting as other self-administered surveys. Nevertheless, self reported information was vulnerable to interpretation, recall, and social desirability bias.

This study’s relatively large sample size is an important strength, providing high power to detect true differences and validate the RS14. The sampling frame provides near universal inclusion of CCIs and a random, representative sample of FBS households, reducing the potential for selection bias and improving generalizability of findings.

This study is the first to examine the validity and reliability of the RS14 in the OSAY population of western Kenya. With psychometric properties with a high level of internal consistency reliability, moderate convergent validity, satisfactory construct validity with a unifactoral solution, this study indicates that the RS14 can be used to measure resilience in this population.

## Supporting information

S1 Data RS14 dataset. This is the dataset used in the investigation of the reliability and validity of the RS14 in orphaned and separated adolescents and youths in western Kenya.
(XLSX)

## Abbreviations

AGFI: Adjusted Goodness-of-Fit Index
CCI: Charitable Children’s Institutes
CDI-S: Child Depression Inventory- Short Form
CFI: Comparative Fit Index
FBS: Family-based care setting
GFI: Goodness-of-Fit Index
MSPSS: Multidimensional Scale of Perceived Social Support
OSAY: Orphaned and separated adolescents and youths
OSCAR: Orphaned and Separated Children’s Assessments Related to Their Health and Well-Being
PCC: Pearson Correlation Coefficient
PHQ-9: 9-item Patient Health Questionnaire
RMSEA: Root Mean Square of Approximation
RS14: Resilience Scale
RS-25: 25-item Resilience Scale
SRMSR: Standardized Root Mean Square Residual
SSA: Sub-Saharan Africa(n)
